# Biophysical properties of the human finger for touch comprehension: influences of ageing and gender

**DOI:** 10.1098/rsos.170321

**Published:** 2017-08-09

**Authors:** A. Abdouni, M. Djaghloul, C. Thieulin, R. Vargiolu, C. Pailler-Mattei, H. Zahouani

**Affiliations:** 1 Laboratoire de Tribologie et Dynamique des Systèmes-LTDS UMR-CNRS 5513, Université de Lyon, ECL-ENISE, 69134 Ecully, France; 2 Laboratoire de Biophysique, Faculté de Pharmacie-ISPB, Université Lyon 1, 69008 Lyon, France

**Keywords:** tactile perception, human finger, ageing, gender, biophysical properties, surface topography

## Abstract

The human finger plays an extremely important role in tactile perception, but little is known about how age and gender affect its biophysical properties and their role in tactile perception. We combined studies on contact characteristics, mechanical properties and surface topography to understand age and gender effects on the human finger. The values obtained regarding contact characteristics (i.e. adhesive force) were significantly higher for women than for men. As for mechanical properties (i.e. Young's modulus *E*), a significant and positive correlation with age was observed and found to be higher for women. A positive correlation was observed between age and the arithmetic mean of surface roughness for men. However, an inverse age effect was highlighted for women. The age and gender effects obtained have never been reported previously in the literature. These results open new perspectives for understanding the weakening of tactile perception across ages and how it differs between men and women.

## 1. Introduction

Human sensing capacities weaken (vision, hearing, taste and smell) over time. A similar decrease is expected to occur for touch sensation and perception. However, studying and interpreting the effects of ageing on touch remain challenging tasks [1]. Over recent years, different studies have focused on understanding tactile perception in different applications such as remote surgery, virtual reality, product design, tactile deficiency detection and rehabilitation, e-shopping and smart surfaces [2–4].

According to the literature, tactile perception is based on the sensory information obtained from the central nervous system. Cutaneous transducers include cutaneous mechanoreceptors, nociceptors (pain) and thermoreceptors (temperature) [5]. They are responsible for detecting sensory stimuli and transmitting them to higher brain structures [6]. Certain studies have focused on touch perception and the influence of age on several morphological and functional features of the peripheral nervous system [5–7]. The source of tactile perception stems from the mechanical activation of sensory receptors located beneath the surface of the skin [8]. The skin has three primary layers [9]: the epidermis, the dermis and the hypodermis. Different types of mechanoreceptors are located in the dermis layer. Touch sensation, pressure, vibrations and cutaneous tension are essentially provided by four major mechanoreceptors: Meissner's corpuscles, Pacinian corpuscles, Merkel's discs and Ruffini's corpuscles [10]. They are sensitive to mechanical deformations which are converted into different specific electrical signals to generate action potentials responsible for transmitting information to the central nervous system [11].

Many studies have illustrated the effects of age and gender on tactile perception. A significant decrease in the concentration of Meissner's corpuscles in the dermis skin layer with ageing has been observed [6,12,13]. In addition, a higher density of Meissner's corpuscles and Merkel discs has been found in the dermis of women's fingers compared with those of men [14,15]. The influences of age and gender have also been highlighted by other studies on vibrotactile detection thresholds (VDTs) and minimum detectable levels of vibration [16]. A significant increase in the threshold as a function of age has been recorded though no gender effect has been observed for the subjects of younger groups regarding vibrotactile sensitivity [16]. However, older women subjects had significantly lower thresholds compared to men [16]. On the other hand, different studies have analysed the reduction in tactile perception with age and differences between men and women based on the characteristics of the finger. The main points investigated in the characteristics of the finger were its size and the density of ridges in the finger print. The results obtained showed higher ridge density for women [14,17]. In addition, the authors reported a direct correlation between finger size and tactile perception. They explained gender effect on tactile perception by finger size and concluded that women have better perception because, statistically, they have smaller fingers than men [15]. Although one study suggested that the changes in mechanical properties of the skin were not related to decreases in tactile discrimination with age [18], we think that a study of the biophysics of the finger could be very beneficial [19]. As the finger is the main tool of tactile perception, a detailed study of the biophysical properties of finger skin could be very helpful in understanding the differences in tactile perception between men and women and its weakening with age. We addressed this issue by studying age and gender effects on the contact properties, mechanical properties and topographical surface properties of the human finger in a cohort of forty subjects of different ages and genders. Various parameters (adhesive force, contact area, Young's modulus, the arithmetic mean of surface topography (SMa)) affecting skin as a function of age and gender were evaluated using different *in vivo* techniques, such as an indentation device, an innovative contactless indentation system and replicas of finger pulp observed with confocal microscopy.

The mechanoreceptors are responsible for tactile perception [20], and their number is the same independent of finger size; however, their density is related to the finger size [15,21]. In conclusion, the contact area defines the number of mechanoreceptors solicited, and it is positively proportional to tactile perception. The contact area is linked to adhesive force, surface topography and mechanical properties. Thus, adhesive force is the dominant parameter as it can increase the contact area [22]. Surface topography is another important parameter as the real contact area is defined by contact between the finger's ridges and the object [14,17]. Finally, account must be taken of mechanical properties as they determine the ability of the finger to deform [22].

In this study, we investigated age and gender effects on the biophysical parameters of the finger directly affecting tactile perception. Our goal was to measure each finger's biophysical parameters in isolation from the other parameters that may affect the results obtained. The indentation test was used to study the contact area by measuring adhesive force. The contactless indentation system was used to understand the finger's mechanical properties by measuring the wave propagation speed, while SMa was proposed to investigate the surface topography of the finger.

## 2. Material and methods

To understand age and gender effects on the properties of the human finger, different techniques have been developed for each of its biophysical parameters. *In vivo* studies on the pulp of 40 subjects (20 of each gender) were performed to characterize the contact, mechanical properties and surface topography of the human finger. The volunteers were divided equally into four groups according to age: G1, 26 ± 3 years; G2, 35 ± 3 years; G3, 45 ± 2 years; G4, 58 ± 6 years. All the subjects were white-collar workers and all the measurements were carried out *in vivo* using non-invasive techniques.

Before starting the measurements, a 30 min stressless acclimatization period was planned in a controlled room (temperature 21 ± 0.5°C; relative humidity 47 ± 5%). Three tests were performed consecutively with each system for each volunteer.

### 2.1. Indentation device

**Figure 1.**
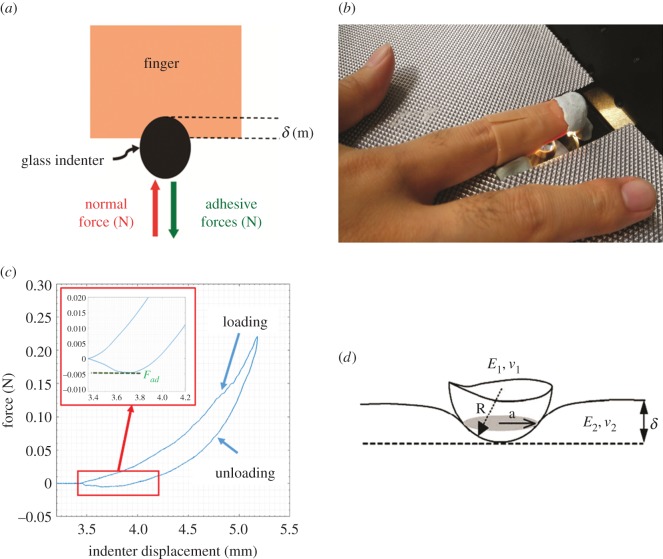
Indentation system. (*a*) Schematic of the indentation system used to analyse the adhesive force between a substrate and a spherical indenter. It is based on applying normal force with an indenter and measuring the response of the skin (adhesive force and penetration depth). *δ*, penetration depth. (*b*) Specific finger indentation device. Different plastic finger guide sizes correspond to the morphology of fingers. They are used to correctly position the finger in order to indent the same predetermined area for all subjects. (*c*) Representative indentation curve on finger skin. The first part of the curve corresponds to the loading phase and the second part to the unloading phase. The maximum adhesive force, *F*_ad_, is the force required to break the contact between the indenter and the finger. (*d*) Elastic contact between soft tissue, a substrate and a solid spherical indenter.

An original light-load indentation device, based on the technique previously developed for cutaneous tissues *in vivo* and *ex vivo* [23,24], has been designed to study finger contact properties (figure 1*a*,*b*). Usually, the indentation test consists of recording the penetration depth, *δ*, of a rigid indenter as a function of the applied normal load, *F*, during a loading/unloading experiment. At the end of each indentation test, the maximum adhesive force (or pull-off force), *F*_ad_, between the spherical indenter and the finger is measured. *F*_ad_ is the force required to break the contact between the indenter and the finger (figure 1*c*). In this study, the indentation tests were specifically performed to measure the maximum adhesive force between the finger and the indenter. The adhesive force reflects the physico-chemical properties of the finger/indenter interface that can influence the contact area between both surfaces and act on touch perception. The contact area between the finger and the indenter was estimated using the JKR theory [22], which is the classical theory used for the indentation of cutaneous tissues [25] (figure 1*d*).

The indentation tests were performed in the controlled displacement mode. The displacement was obtained from a displacement table and controlled by a displacement sensor. All the indentation tests were carried out at constant indentation speed *V* = 0.5 mm s^−1^ and under a maximal applied normal load *F* = 0.2 N. These experimental conditions are widely observed in the literature to estimate finger mechanical properties and to evaluate problems of touch perception [4,26]. The indenter used was a spherical glass indenter with a radius of curvature *R* = 2.5 mm.

### 2.2. Air blast system

A contactless indentation system based on techniques used in ophthalmology was proposed [27]. The principle of the test is to subject the finger to a direct blast of air on a predetermined area (1 × 2 cm) and measure the deformation with a linear laser (Keyence 7020) [28]. The advantage of this test is that the skin returns freely into position without any external influence and it avoids the adhesive effect previously observed for the human skin [29]. The device developed makes it possible to carry out tests of short duration such as impact tests (4 ms) using air blasts. In addition, the pressure used for the air blast is controlled. Finally, the deformation of the skin observed with a linear laser permits measuring the velocity of the shear wave generated and then calculating the Young's modulus.

**Figure 2.**
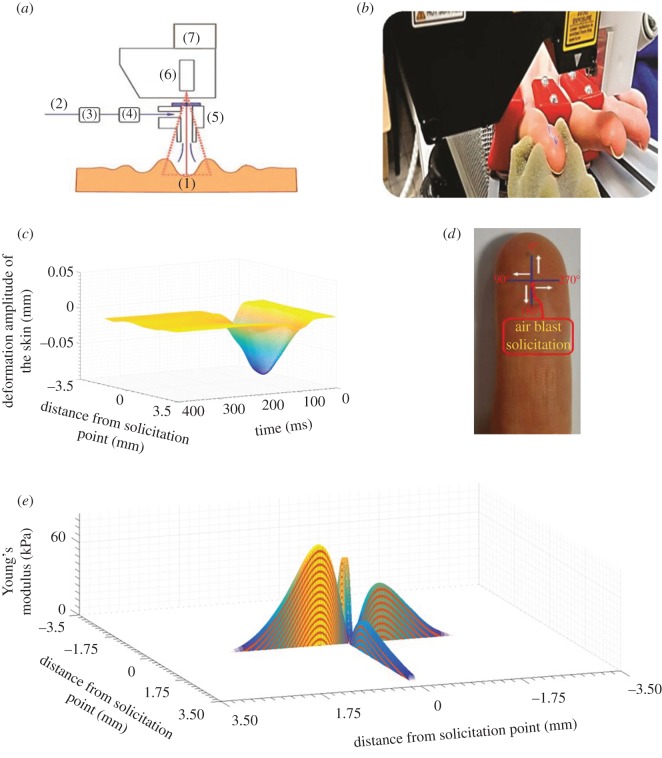
Contactless indentation system concept. (*a*) Principle of the contactless indentation system; (1) skin, (2) air compressor, (3) pressure regulator, (4) solenoid valve, (5) glass pipette, (6) 2D linear laser, (7) turntable. (*b*) Photograph of the contactless indentation system with the finger fixation technique. (*c*) Skin deformation measured by the linear laser. The deformation amplitude is plotted as a function of time (milliseconds) and the distance from the point solicited (millimetres). (*d*) The predetermined directions for studying the anisotropy of mechanical properties. (*e*) Three-dimensional anisotropy effect of Young's modulus as a function of the distance from the point solicited (millimetres) in four directions.

A detailed diagram of the contactless indentation device is presented in figure 2*a*. An air compressor was used to provide an air flow controlled by a pressure regulator. The duration of the air flow output was controlled by solenoid valves. The air passes through a glass pipette with an inner diameter of 1 mm. The distance between the end of the pipette and the sample was fixed at 10 mm. A laser system was used to measure the vertical displacement of the skin during the test with a sampling frequency of 8 kHz, as shown in figure 2*b*,*c*. The laser line was composed of 400 sensors over a length of 7 mm. A software application was developed with Labview^TM^ language (National Instruments) to control the system. A turntable was placed on the head of the laser to rotate both the laser and the glass pipette together around the pipette axis. Centring the glass pipette on the laser line permitted studying the anisotropy of the finger's mechanical properties, as shown in figure 2*d*,*e*. A soft support was used to fix the finger during the measurements. This technique did not apply any extra forces on the finger. The hand immobilizer (KLS Martin) developed especially for hand surgery is used. It ensured the fixation and natural extension of the fingers.

Impact tests with an air flux of 3 bars for 4 ms were carried out on a predetermined area of the finger. The response of the skin during the solicitation and its free return can be seen in figure 2*c*. The measurements were performed in different directions (body direction and normal direction), as can be observed in figure 2*d*. To obtain robust results, three tests were performed for each measurement and then the mean and standard deviation were calculated.

A typical response of the finger pulp to impact tests is presented (figure 2*c*). Owing to the impact, the local deformation of the finger pulp generated the propagation of an offset surface displacement. Tracking the deformation phenomena along the laser line permitted calculating the speed of wave propagation using equation (4) in table 1. The velocity of the shear wave *c* was calculated as the distance between the sensors of the laser line (sensor positions)/the corresponding time. The Young's modulus was evaluated with equation (5) and equation (6) in table 1; $E=2\rho (1+v)c^{2}$ [30,31].

**Table 1.** List of the mathematical equations used.

equation	description	equation no.
$F_{\mathrm{Elastic}}=\frac{4}{3}E^{*}\sqrt{R}\delta^{3/2}$	where E* is the reduced elastic modulus expressing contact; *R* is the indenter's radius; and *δ* is the penetration depth.	(1)
$\frac{1}{E^{*}}=\frac{1-\nu_{1}^{2}}{E_{1}}+\frac{1-\nu_{2}^{2}}{E_{2}}$	where E* is the reduced elastic modulus expressing contact; *E*_1_, *E*_2_, are the elastic modulus of indenter and substrate, respectively; *ν*_1_, *ν*_2_ are Poisson's ratios.	(2)
$\alpha =\sqrt[{3}]{\frac{3}{4}\frac{R}{\overset{*}{E}}(F_{N}+2F_{\mathrm{ad}}+2\sqrt{F_{\mathrm{ad}}(F_{N}+F_{\mathrm{ad}})}}$	where *α* is the corrected contact radius; *R* is the radius of curvature of the spherical punch; *F*_N_ is the normal force; *F*_ad_ is the adhesive force; E* is the reduced elastic modulus; *E* is the elastic modulus calculated by the wave propagation speed.	(3)
$\overset{*}{E}=\frac{E}{1-\nu^{2}}$		
$\frac{\partial^{2}u}{\partial x^{2}}=\frac{\partial^{2}u}{c^{2}\partial t^{2}}$	where *u* is the displacement vector; *c* is the wave propagation speed.	(4)
$c=\sqrt{\frac{G}{\rho }}$	where *G* is the shear modulus and *ρ* ≈ 1000 kg m^−3^for the skin [33].	(5)
$G=\frac{E}{2(1+\nu )}$	where *E* is the Young's modulus; *ν* = 0.5 is Poisson's ratio [33].	(6)
$\Psi_{b,a}(x,y)=\frac{1}{\sqrt{a_{x}a_{y}}}\Psi \left(\frac{x-b_{x}}{a_{x}},\frac{y-b_{y}}{a_{y}}\right)$	where *a_x_*, *a_y_* are the contraction coefficients; *b_x_*, *b_y_* are the translation coefficients.	(7)
$W_{\Psi }^{f}=f(x,y)⊗\frac{1}{\sqrt{a_{x}a_{y}}}\Psi_{b,a}(x,y)$	where *f*(*x, y*) denotes the topographic surface and $W_{\Psi }^{f}$ the transformed surface.	(8)
$\text{SMA}(a)=\sum_{x=1}^{M}\sum_{y=1}^{N}\frac{|f_{a}(x,y)|}{MN}$	where SMa is the arithmetic mean of the surface topography at each scale; *M* and *N* are surface dimensions.	(9)

The Young's modulus reflects the intrinsic property of the material; it is linked to the evaluation of a rheological model. In our study, the Kelvin–Voigt rheological model was used to estimate the relationship between the propagation velocity and the Young's modulus [32]. The tracking algorithm developed was used to obtain the wave propagation speed for each pair of sensors, corresponding to a Young's modulus. The mean Young's modulus of the finger was calculated from the point of solicitation to the end of the laser line (figure 2*e*). The contact area was calculated based on the Young's modulus computed and the adhesive force measured by the indentation system, see equation (3) in table 1.

### 2.3. Confocal chromatic imaging

**Figure 3.**
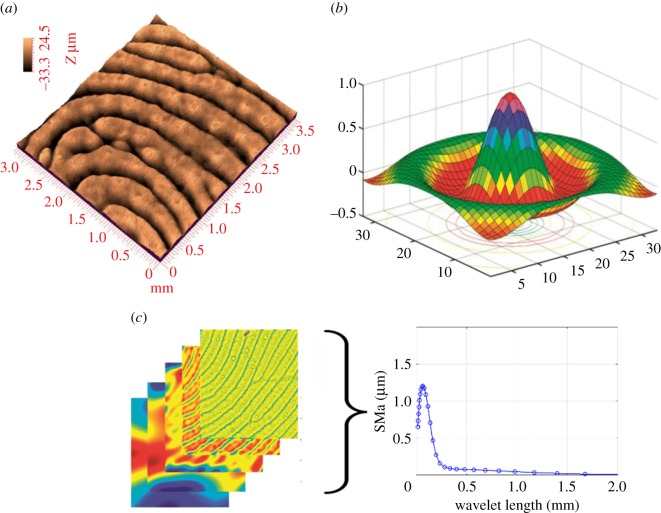
Surface topography analysis. (*a*) Fingerprint scanned replica by confocal chromatic microscopy. Four by four millimetre scanned zone in the predetermined area (1 × 2 cm) (*b*) Mexican hat wavelet used to analyse the image. (*c*) The wavelet decomposition of the scanned image and the arithmetic mean of surface topography at wavelet length.

Studying the surface topography of human fingers provides an understanding of the age and gender effects on fingerprint roughness characteristics. Negative silicon replicas of finger pulp were made immediately after performing the finger mechanical property measurements. To obtain the negative silicon replicas, a liquid elastomer (Silflo^®^, Flexico Ltd, UK) used for medical applications mixed with a catalyst was spread onto the same predetermined area (1 × 2 cm) as used in the previous measurements. After a few minutes, the elastomer hardened and the negative of the skin relief was obtained [33]. To reconstruct a three-dimensional image of the cutaneous relief, a white light interferometric device was used [34,35]. Confocal chromatic imaging provides non-contact, reliable, accurate and reproducible dimensional measurements with extremely high resolution (figure 3*a*). This system was used to obtain a high-precision positive image of the cutaneous relief.

To study age and gender effects on topographic properties, multiscale roughness characterization was performed by applying a continuous wavelet transform. The principle of wavelet analysis is based on the convolution of the topographic surface with wavelets of variable dilatations and translations of the mother wavelet *ψ*(*t*) (see equation (7) and equation (8) in table 1. The Mexican hat was used as the mother wavelet (figure 3*b*). This method was used to perform the multi-resolution study of the topographic surface (figure 3*c*) [36].

One of the important points of continuous wavelet decomposition is that high-resolution wavelets are used to study the scales of the signal analysed in all wavelength bands. This property was used to quantify the roughness criterion for each decomposed scale. The result is represented by a spectrum of a roughness criterion determined for all scales, i.e. a multiscale signature with a given number of scales: 32.

After determining the decomposition of the surface, the method consists of quantifying the SMa at each scale (figure 3*c*; equation (9) in table 1). *M* and *N* are the surface dimensions in the *x-* and *y-*directions, respectively (see equation (9) in table 1) [37].

### 2.4. Statistical analyses

Matlab software was used for statistical data analysis. Analysis of variance (ANOVA) is a statistical model used to analyse the differences between and among groups and to determine if the differences between the means are statistically significant. The data are considered statistically significant if the *p*-value is lower than the significance level defined (0.05 in our case). In our study, we used ANOVA to see whether we had statistically significant differences as a function of age and gender (adhesive force, Young's modulus and SMa).

In statistics, the Pearson correlation coefficient (*r*) is a measure of the linear dependence (correlation) between variables. The Pearson correlation coefficient value was used to determine the strength of the relationship between variables. The correlation is considered strong for an *r*-value higher than 0.8. In this study, we used the *r* coefficient to determine whether we had a statistically linear correlation between age groups regarding mechanical properties [38].

## 3. Results

Substantial differences between the finger properties of women and men and as a function of age were encountered. The results are described in the following sections, which successively deal with the finger's contact properties, mechanical properties and topographic characterization as a function of age and gender.

### 3.1. Adhesive force

**Figure 4.**
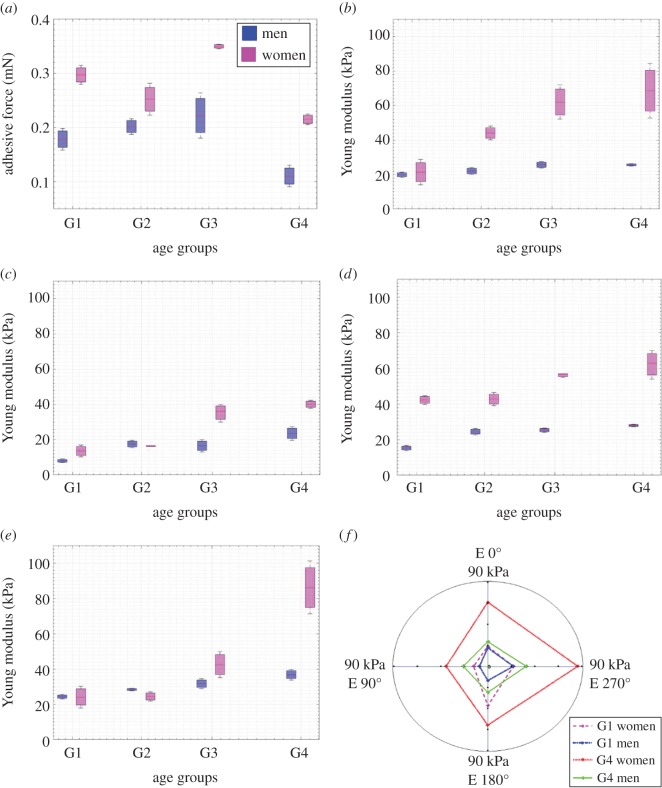
Age and gender effects on biophysical properties. (*a*) Adhesive force and (*b*–*e*) Young's modulus in 0°, 90°, 180° and 270° directions. (*f*) Anisotropy of finger mechanical properties as a function of directions for G1 and G4. (*a–e*) ANOVA and Pearson's statistical analyses: *r* < 0.8, *p *< 0.05; *r* > 0.8, *p *< 0.05; *r* > 0.8, *p *< 0.05; *r* > 0.8, *p *< 0.05, respectively. (*r* > 0.8 highly correlated, *p *< 0.05 statistically significant).

Concerning the finger's contact properties, the adhesive force measured for men and women as a function of age is presented in figure 4*a*. The results show a very clear gender effect with higher adhesive forces for women. The adhesive force varies from 0.1 to 0.25 mN for men and between 0.22 and 0.36 mN for women. The difference in adhesive force between women and men can be considered very significant for each age group. However, the indentation test measurements do not signal a clear age effect for either men or women. Indeed, the adhesive force varies nonlinearly as a function of age. The finger's contact properties and adhesive force studied as a function of age and gender effects have never been reported in the literature up to now.

### 3.2. Mechanical properties and anisotropy effect

Figure 4*b*–*e* highlights the Young's modulus of the finger measured in four predefined directions as a function of age and gender. The results provide insight on age and gender effects on the finger's mechanical properties. The Young's modulus, *E*, is significantly and positively correlated to age (*p* < 0.05, *r* > 0.8 for the 0° direction) independently of the gender effect. The Pearson correlation coefficient shows higher values than 0.8 in all directions; therefore, a linear correlation can be observed as a function of age. In addition, the evolution of Young's modulus with age is anisotropic as a function of the direction measured. The results demonstrate a higher *E* for the exterior part of the finger skin (0°, 270°). The exterior part of the finger is more exposed to the environment and to repeated friction in daily life (i.e. writing); therefore, increasing anisotropy of mechanical properties with age can be observed (figure 4*f*). Figure 4*f* shows the anisotropy of the finger's mechanical properties for both men and women of the youngest and the older groups. The linear correlation (*r* > 0.8) of Young's modulus and age means that the same anisotropy effect can be concluded for the other age groups. To the best of our knowledge, the effects of age on the finger's mechanical properties have never been reported previously.

Clearly, women generally have a higher Young's modulus than men. Figure 4*b*,*e* demonstrates a clear gender effect with age where the difference of mechanical properties between men and women is greater for the older groups. The results obtained are consistent with previous findings which indicated that women have a higher Young's modulus [4,26]. These authors studied the gender effect on the mechanical properties of the finger using an indentation system. The indentation method has certain limits, such as the effect of adhesive force on the Young's modulus calculated, which considerably modifies the indenter/material contact area and significantly disturbs the estimation of the Young's modulus. In addition, they studied the gender effect on only one age group (34–56 years [4] and 25–33 years [26]). In this study, we avoided the limitations of the systems reported in the literature. In conclusion, we obtained the same gender effects on finger mechanical properties as those already reported in the literature. However, we carried out a full study of the gender effect of finger mechanical properties, without the limitations of classical systems.

### 3.3. Contact area

**Figure 5.**
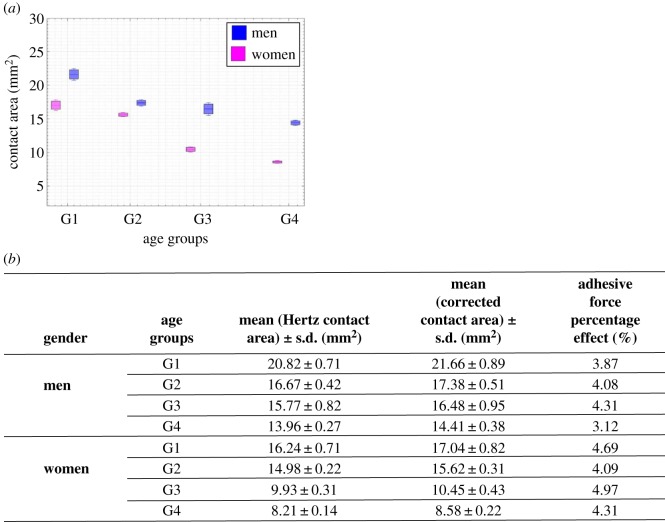
Age and gender effects on contact area properties. (*a*) Indenter/finger contact area. ANOVA and Pearson's statistical analyses: |r|≫0.8, p≪0.05 (*r* > 0.8 highly correlated, *p *< 0.05 statistically significant). (*b*) Contact area results as a function of age, gender and adhesive force. The table gives the percentage of correction due to adhesive force with JKR, equation (3) in table 1.

Figure 5*a* highlights the contact area of the finger corrected by JKR theory [22], and calculated using the adhesive force and the mean Young's modulus as a function of age and gender. The correction percentage of the contact area demonstrates the effect of the adhesive force. The contact area clearly reduces with age for both men and women (figure 5*a*,*b*) and this reduction is significantly greater for men than for women with age p≪0.001. The results show a high correlation between age and contact area reduction *r* = −0.99 for both men and women. The findings obtained mainly highlight age and gender effects on Young's modulus.

### 3.4. Surface topography

**Figure 6.**
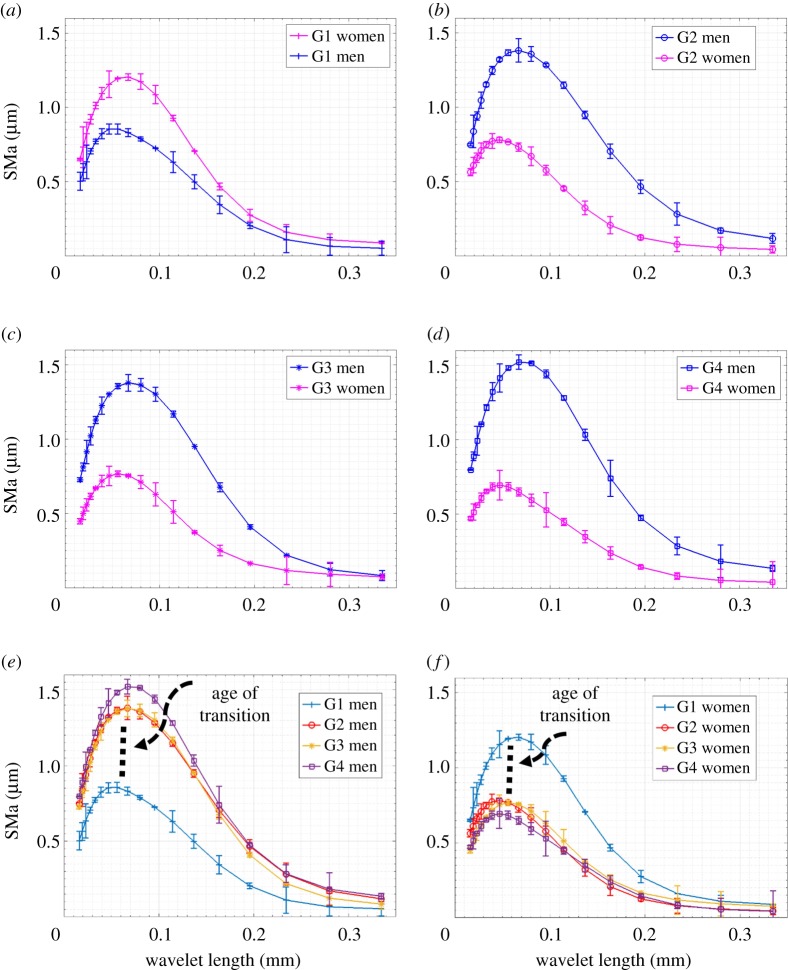
Arithmetic mean of surface topography as a function of age and gender. (*a*–*d*) SMa as a function of wavelet length for G1, G2, G3 and G4. (*e*–*f*) Age effect on SMa for men and women, respectively. ANOVA statistical analyses: (*a*–*d*) p≪0.05 for all age groups; for (*e*–*f*) p≪0.05 between G1 and other groups and *p *> 0.05 between G2, G3 and G4 (*p *< 0.05 statistically significant).

These measurements permitted studying age and gender effects on fingerprint topography. Figure 6*a*,*e* shows the gender effect on the mean roughness spectrum SMa with the standard deviation for each age group. A clear difference in roughness amplitude can be observed between men and women for all age groups. From the second to the fourth group, the roughness amplitude of SMa is higher for men than for women. Regarding the first age group, an inverse gender effect compared with the other age groups can be observed. The maximum amplitude of SMa decreased from 1.2 to 0.69 µm for women and increased from 0.85 to 1.52 µm for men (table 2). On the other hand, all the maximum amplitudes of SMa for all the age groups of men and women are presented within a range of wavelet lengths from 0.047 to 0.067 mm.

**Table 2.** SMa results as a function of age and gender. The table presents the maximum SMa amplitude and corresponding wavelet length.

		G1	G2	G3	G4
men	wavelet length of max SMa (mm)	0.056	0.067	0.067	0.067
	max SMa (µm)	0.85 ± 0.03	1.38 ± 0.08	1.38 ± 0.05	1.52 ± 0.05
women	wavelet length of max SMa (mm)	0.056	0.047	0.056	0.046
	max SMa (µm)	1.2 ± 0.02	0.78 ± 0.01	0.77 ± 0.02	0.69 ± 0.07

To understand the results obtained, we propose separate studies for age and gender effects on the topography properties of the human finger. Generally, the corresponding wavelet length to the maximum SMa amplitude was higher for men for all the age groups (figure 6*a*,*d* and table 2). Although the SMa provides the distribution of the pattern in the image and the wavelet length analysed, it is inversely proportional to its repeatability. This means higher repeatability for short wavelet lengths. The results obtained correspond to what can be found in the literature on the gender effect on ridge density. Different studies showed higher ridge density for the index finger for women than men [14,17,39]. Previous studies are consistent with our findings regarding the gender effects revealed by surface topography.

Figure 6*e*,*f* clearly shows the pronounced effect of age and the evolution of SMa amplitudes with age for both men and women. The topographical properties evolved considerably between the first and second age groups (25 years old and 35 years old, respectively), and then changed slightly for the subsequent age groups. This can be explained by the transition age effect. The transition age in skin topography properties has already been mentioned in the literature for the forearm and is about 37 years old [40]. Therefore, the results obtained for the age effect on SMa can be explained by the age of the transition effect. This phenomenon occurs at 30 ± 2 years old for the finger according to our results.

## 4. Discussion

Adhesive force and mechanical properties are the most important parameters that affect the indenter/finger contact area (figure 5*b*). The results obtained showed a higher contact area calculated for men (figure 5*a*). However, the real contact area is that between the fingerprint ridges and the object. The literature includes works relating to the gender effect on ridge density. Higher ridge density was found for women [14,17]. Our results show a higher contact area calculated for the same normal force for men but other parameters should be taken into consideration, such as mechanoreceptor and ridge density in the finger, in order to understand the effect of gender on tactile perception [21]. The results obtained for adhesive force might be explained by the higher density of eccrine sweat glands in the hand for women. Indeed, the density of sweat glands in the hands is around 600 glands cm^−2^, whereas it is around 200 glands cm^−2^ on the rest of the body [41,42]. The sweat glands secrete a solution mainly composed of water and electrolytes at the origin of the adhesion phenomena, due to capillary force, observed between the finger and the indenter surface. Pores are the small openings on the surface of fingerprint ridges formed by the ducts of sweat glands [43]. As mentioned in the literature [15], women have a higher pore density on their fingerprints. In conclusion, the higher adhesive force for women observed in the results can be explained by higher fingerprint pore density related to the density of sweat glands.

Certain studies in the literature investigated the gender effect on finger size. They reported that, statistically, men's fingers are bigger than women's fingers [15]. However, other studies have shown the age effect on skin and demonstrated a reduction in skin thickness as a function of age [44]. Moreover, the loss of elastin fibres and collagen fibres leads to wrinkles and topographical changes [33]. The anatomy of the finger shows the absence of muscles and that it is composed of nail, skin and bones. The lack of information in the literature on finger characteristics leads us to propose hypotheses based on our experimental results.

For women, the finger's Young's modulus increases significantly with age (table 3). We explain this effect by the difference in finger size between men and women, and skin thickness reduction with age. As the skin's thickness reduces with age, the effect of bone (a very hard organ) on the mechanical properties of finger pulp probably increases. The results obtained for the Young's modulus in women prove our hypothesis (table 3).

**Table 3.** Trends of age and gender effects on finger biophysical properties. The table summarizes age and gender effects on the biophysical properties of the human finger. (*r* > 0.8 highly correlated, *p* < 0.05 statistically significant).

			age effect	
biophysical properties		gender effect	men	women
contact properties	adhesive force	significantly higher for women	no linear correlation	no linear correlation
	contact area	significantly higher for women	decrease	decrease
mechanical properties	Young's modulus	significantly higher for women	increase	increase
topography properties	SMa	G1: significantly higher for women	increase	decrease
		Other groups: significantly higher for men		

With regard to the men's results, the finger's Young's modulus increases slightly as a function of age (table 3). This effect could be explained by the reduction in skin thickness similar to that of women. However, the increase is not as high as that found in the results obtained for women, which can be explained by the size effect on the finger. As men's fingers are bigger, their finger skin remains thicker than that of women. Furthermore, the bone is still slightly farther from the skin than for women and its solicitation is less pronounced. Therefore, the Young's modulus is slightly higher as a function of age for men.

In general, people often develop calluses on the fingers used, mostly of the dominant hand, due to writing with pens and pencils. Calluses can develop on any part of the skin exposed to friction over a long time. This phenomenon is a physiological mechanism that protects underlying soft tissues from mechanical stress by increasing the thickness of the stratum corneum (SC) [45,46]. In addition, the barrier created reduces the loss of water and other materials from the body [47].

For men, the increase of SMa with age could be explained by the effect of callosity and the increased thickness of the SC. Callosity increases skin roughness, which means greater amplitude in the skin's relief [45]. On the other hand, the effect of callosity increases with time due to the repeated use of the hands, resulting in the augmentation of SMa with age (see tables 2 and 3).

Generally, women attach more importance to taking care of their skin and regularly use cosmetic products [48]. The latter may limit damage to the skin from daily interaction with the environment, and in particular the effect of callosity. The results obtained for adhesive force prove our hypothesis (table 3). The higher adhesive force observed for women in all the age categories studied is due to a higher amount of sweat and water loss than men [25]. Higher water loss could lead to dryness in the finger with time, flattening skin reliefs. In conclusion, the decrease in SMa with age for women could be due to a higher amount of sweat and water loss over time.

## 5. Conclusion

This study provided *in vivo* measurements of 40 human fingers. Different innovative systems were developed to characterize finger biophysical properties related to age and gender. Three different concepts were developed to understand these age and gender effects. Firstly, contact properties and adhesive force as a function of age and gender were studied using an indentation system. Secondly, an innovative contactless system was adapted to measure the mechanical properties of finger pads. A study of age and gender effects based on the Young's modulus measured was performed. The adhesive force and Young's modulus results obtained were used to calculate and correct the indenter/finger contact area. The latter was investigated in order to understand the importance of each parameter in tactile perception. In addition, this study helped to comprehend age and gender influences on tactile perception. Thirdly, a multiscale study with wavelet transforms based on replicas of the finger pulp was carried out. This provided insight on age and gender effects on the arithmetic mean SMa of the surface topography at each scale. These results revealed clear trends in age and gender effects on the biophysical properties of fingers. The results showed that the modifications of the finger's skin properties due to ageing and gender differ compared to other body locations such as the forearm.

To the best of our knowledge, the techniques used and the results of the analysis of age and gender effects obtained from a panel of 40 subjects have never been reported up to now. These are our first findings and further studies will be described in forthcoming papers.

## Data accessibility

The authors are unable to make the full dataset and algorithms associated with the work available at the present time for two reasons. Firstly, to do so would risk waiving the anonymity of the individual volunteers involved in the study, who volunteered on the basis of retaining anonymity. Secondly, among its other functions, the Agence Nationale de la Recherche encourages patent applications from completed work it has funded and supported—there are currently a number of patent applications under consideration relating to the dataset generated and algorithms used in this study. On the grounds that to provide access to these materials would invalidate the patent application, we have requested that the Editor grant a waiver for the normal data accessibility requirements. Should readers wish to access the dataset and algorithms, this is possible on a case-by-case basis by contacting Abdenaceur Abdouni (abde-naceur.abdouni@doctorant.ec-lyon.fr or abdouninaceur@hotmail.com).
